# Incidence and determinants of neonatal near miss in south Ethiopia: a prospective cohort study

**DOI:** 10.1186/s12884-020-03049-w

**Published:** 2020-06-09

**Authors:** Tesfalidet Tekelab, Catherine Chojenta, Roger Smith, Deborah Loxton

**Affiliations:** 1 Research Centre for Generational Health and Ageing, Faculty of Health and Medicine, University of Newcastle, Newcastle, Australia; 2 College of Medical and Health Sciences, Wollega University, Nekemte, Oromia Ethiopia; 3 The Mothers and Babies Research Centre at the Hunter Medical Research Institute, University of Newcastle, Newcastle, Australia

**Keywords:** Neonatal near miss, Neonatal deaths, Severe maternal complication, Ethiopia

## Abstract

**Background:**

For every neonate who dies, many others experience a near miss event that could have but did not result in death. Neonatal near miss is three to eight times more frequent than neonatal deaths and, therefore, is more useful for assessing the determinants of adverse neonatal outcomes. The aim of this study was to assess the incidence and determinants of neonatal near miss in south Ethiopia.

**Methods:**

A facility-based prospective study was conducted among 2704 neonates between 12 July to 26 November 2018. The neonates were followed from the time of admission to hospital discharge or seven postpartum days if the newborn stayed in the hospital. The data were collected by interviewer-administered questionnaire and medical record review. Logistic regression was employed to identify the distant, intermediate and proximal factors associated with neonatal near miss. The independent variables were analysed in three hierarchical blocks. Adjusted odds ratios (AORs) and 95% confidence intervals (CIs) were used to determine the strength of the associations.

**Results:**

The incidences of neonatal near miss and neonatal death were 45.1 (95% CI = 37.7–53.8) and 17.4 (95% CI = 13.0–23.3) per 1000 live births, respectively. Of those newborns who experienced neonatal near miss, more than half (59.8%) of their mothers were referred from other health facilities. After adjusting for potential confounders, the odds of neonatal near miss were significantly higher among neonates with a low monthly income (< 79 USD monthly), a birth interval of less than 24 months and where severe maternal complications had occurred.

**Conclusion:**

Strategies to improve neonatal survival need a multifaceted approach that includes socio-economic and health-related factors. The findings of this study highlight important implications for policymakers with regard to neonatal near miss. In particular, addressing inequalities by increasing women’s income, promoting an optimal birth interval of 24 months or above through postpartum family planning, and preventing maternal complications may improve newborn survival.

## Background

Around the world, remarkable progress has been made regarding the reduction of child mortality. However, child survival remains an urgent concern [1, 2]. Globally, in 2017, 5.4 million children died before reaching their fifth birthday and about half (47%) of these deaths occurred in the first month of life [3, 4]. Worldwide, the neonatal mortality rate (NMR) fell by 51%, from 37 deaths per 1000 live births in 1990 to 18 per 1000 live births in 2017. The reduction in NMR was less than that of children aged one to 59 months, which dropped by 63% from 2000 to 2017. Sub-Saharan Africa had the highest NMR at 27 deaths per 1000 live births in 2017 [3]. By the end of 2030, the Sustainable Development Goal (SDG) global target is an NMR of 12 deaths per 1000 live births. If the slow reduction in neonatal death continues, the proportion of neonatal deaths in children under five years of age will actually increase, from 47 to 52% by 2030 [2, 5].

For every newborn who dies, many others develop severe complications. A systematic review by Santos (2015) states that neonatal mortality is three to eight times less frequent than neonatal near miss (NNM) events [6]. Near miss is becoming increasingly accepted as a tool to evaluate and improve the quality of maternal health care [7, 8]. Near miss are important because they follow a similar path to deaths and so provide numerous cases for analysis. It has been assumed that this idea could also be useful in the neonatal context, to improve newborn survival [9–12]. However, to date, there is no standardised definition or internationally agreed identification criteria for NNM cases. One definition is that an NNM case refers to “a neonate who nearly died but survived a severe complication that occurred during the neonatal period” [9].

Ethiopia has shown a reduction in the under-five mortality rate by two-thirds within 22 years (1990 to 2012), from 204 to 68 deaths per 1000 live births and has achieved the UN’s Millennium Development Goal 4 ahead of time [13, 14]. However, the change in NMR is not as substantial as the change in post neonatal and child mortality, which is still high at 29 per 1000 live births [14]. In Ethiopia, neonatal deaths account for 44% of under-five deaths [15]. According to the 2016 Ethiopian Demographic and Health Survey (EDHS) report, the NMR in Ethiopia declined from 49 deaths per 1000 live births in 2000 to 29 deaths per 1000 live births in 2016, a reduction of 41% over the last 16 years [14]. Studies conducted in Southwest and Northern Ethiopia reported NMRs 35.3 and 62.5 deaths per 1000 live births, respectively [16, 17]. A possible explanation for the difference in these results could be that the study conducted in Northern Ethiopia collected data both from health facilities and the community, unlike the Southwest study that included data from the community.

Globally, only three studies have assessed risk factors for NNM cases [18–20]. Prospective hospital-based studies in Brazil revealed that lack of antenatal care (ANC), less than the recommended number of ANC visits, maternal complications and caesarean section were associated with increased risk of NNM [18, 19]. A study by de Lima stated that maternal complications had no association with NNM but that previous caesarean section and a maternal age of older than 35 years were associated with lower NNM [19]. Studies have examined risk factors associated with neonatal mortality and adverse perinatal outcomes in Ethiopia but to the authors’ knowledge, there have been no studies examining NNM [17, 21–23]. A study conducted in Addis Ababa, Ethiopia reported that maternal complications were strong predictors of adverse perinatal outcomes such as low birth weight, neonatal death, low Apgar score and birth asphyxia [23]. Assessing the incidence and determinants of NNM is important for policymakers in that it provides evidence that helps to improve perinatal health outcomes. This study assessed newborn outcomes up to hospital discharge or seven postpartum days if the newborn stayed in the hospital. The aim of this study is to assess the incidence and determinants of NNM in south Ethiopia.

## Methods

### Study settings and design

This was a prospective cohort study involving pregnant women who were admitted for delivery or pregnancy-related complications and their newborns. The study was conducted between 12 July to 26 November 2018 at three selected hospitals in the Southern Nations, Nationalities and Peoples’ Region (SNNPR), Ethiopia. The SNNPR is located in the south part of Ethiopia and its capital city is Hawassa, which is located 285 km from Addis Ababa. According to the Central Statistical Agency of Ethiopia (CSA), the region has a total population of 15 million, of whom 50.3% are women [24]. According to the Regional Health Bureau’s 2014 annual report, the region has 21 governmental hospitals [25].

The hospitals selected for the study were Hawassa University Comprehensive Specialized Hospital (HUCSH), Nigist Eleni Mohammed General hospital (NEMGH) and Durame General Hospital (DGH). HUCSH is located in Hawassa, the capital city of the SNNPR. HUCSH is a teaching referral hospital ranked in the top level of the three-tier Ethiopian health care system; it provides services for more than 18 million people. The hospital has over 400 beds for inpatient services and three operation rooms for obstetric and gynaecological surgeries. More than 4000 deliveries per year are conducted in the hospital, of which 30 to 35% are caesarean section. NEMGH is a general hospital located in Hossana city that provides services to a catchment population of 1,506,733. The hospital has 192 beds for inpatient services and oversees 6643 deliveries annually. The DGH is located in Durame city and serves as a referral hospital for the Kembata Tembaro zone. The hospital provides services to 1,100,000 people and oversees 1700 deliveries annually. At the time of this study, each hospital has a neonatal intensive care unit which provides specialty care for the newborns. However, the teaching hospital (HUCSH) is well equipped and has a higher number of paediatrician/neonatologists than the regional hospitals.

### Sample size

Assuming an incidence ratio of early neonatal death of 369 per 2142 deliveries [26], a 95% CI, a margin of error 2%, a design effect of two and a non-response rate of 10%, the final sample size was 3010.

### Data collection tool and techniques

A template of the tool was prepared using an online survey application *(Survey Gizmo)* and downloaded to iPads for offline data collection. Three trained data collectors were recruited to collect the data. Data were collected on a daily basis. Using an interviewer-administered questionnaire and medical record review, data were collected on sociodemographic characteristics, reproductive characteristics, pregnancy complications, and pregnancy outcomes until the time of discharge from the hospital or seven postpartum days. In cases in which readmission occurred, only the previously collected data were retained for analysis. The criteria to identify the NNM were developed by reviewing literature [10, 19, 26, 27] that used pragmatic criteria (Apgar score at five minutes less than seven, gestational age based on last menstrual period or ultrasound less than 33 weeks, birth weight less than 1750 g), clinical criteria (cyanosis, respiratory rate > 70 bpm, absence of regular breathing, cardiac arrest, persistent bradycardia < 80 bpm, visible jaundice in first 24 h, persistent tachycardia > 200 bpm, seizures, inability to suck within 12 h or inability to suck with six attempts without pause, any active non-traumatic bleeding, haematuria, anuria > 24 h, apathy, poor tolerance of feeds within 12 h, abdominal distension and vomiting) and management criteria (any intubation, cardiopulmonary resuscitation, use of vasoactive drugs, blood transfusion (simple blood transfusion and exchange blood transfusion), use of anticonvulsant, phototherapy, parenteral antibiotics). The neonates were assessed for the entire period of hospital stay. There was no follow-up after the hospital discharge or seventh postpartum day. Therefore, data refer only to intra-hospital NNM and neonatal mortality.

### Study variables

The conceptual framework to address the NNM adapted by reviewing literature based on a model for NNM and neonatal death [18, 19, 28, 29]. According to the framework, NNM is affected by various factors. The factors are grouped into three categories: The first includes distant factors: sociodemographic and economic status determined by factors such as age of the mother, maternal and paternal education, maternal and paternal occupation, income, marital status and place of residence. The second category includes intermediate factors: birth interval, pregnancy intention, number of children, ANC utilisation, and frequency of ANC utilisation and referral status. The third includes proximal factors: mode of delivery, sex of the newborn and maternal complication. Table 1 describes the variables used in the analysis. NNM was the dependent variable, which was coded as 0 for “no” and 1 for “yes”.

**Table 1:** Description of variables included in the analysis in south Ethiopia, 2018

Variables	Description and categorization
**Distant**	
Place of residence	The usual place of residence where the woman lives. Rural was coded as ‘1’ and Urban was coded as ‘2’.
Parental Education	Maternal and paternal education status. Categorized into two groups as ‘no formal education and ‘primary [1–8]’ and ‘Secondary [9–12]’ and above’.
Parental Occupation	Maternal and paternal occupation. Classified as ‘housewife for mothers, ‘farmer’ for fathers and ‘others’.
Monthly income	Income received by family in each month. Categorized into four based on lowest 25 percentile; <=79USD, 80-121USD, 122-155USD and > 155USD.
**Intermediate**	
Birth interval	The period between last pregnancy and current pregnancy in months. Categorized as less than 24 months and greater than or equal to 24 months.
Type of pregnancy	Intention to become pregnant when women conceived. Grouped as ‘planned and unplanned pregnancy’
ANC utilization	Whether the women received at least one antenatal care check-up during pregnancy by skilled health care personnel. (1-Yes, 2-No)
Frequency of ANC	Number of visits for pregnancy check-up (1–1-3 visits, 2–4 visits).
Women refereed for delivery	Refereed from other health facility for delivery or pregnancy complication (1-Yes, 2-No)
**Proximal**	
Mode of delivery	Categorized as “1-Vaginal delivery, 2- Caesarean section
Sex	The sex of the neonate (1- Female, 2- Male)
Maternal complication*	The occurrence of potentially life-threatening condition to women (1-Yes, 2-No)

* Potentially life-threatening condition include severe postpartum haemorrhage, severe-preeclampsia, eclampsia, uterine rupture, sepsis

### Data processing and analysis

Data were exported from *Survey Gizmo* into SPSS software version 20 for analysis. Descriptive statistics of neonatal near miss rate (NNMR), NMR, severe neonatal outcomes (SNO), and severe neonatal outcome rate (SNOR) and mortality index (MI) were calculated. NNMR was calculated as the number of NNM cases per 1000 live births. SNO is the sum of NNM and neonatal deaths. SNOR refers to the number of neonates with SNO per 1000 live births. The mortality index is calculated as the number of neonatal deaths divided by the number of neonates with SNO [10, 19, 30]. The analysis of the determinants of NNM was restricted to singleton live-born neonates since multiple pregnancies have higher odds of newborn morbidity associated with prematurity and pregnancy complications [22, 31, 32].

Variance inflation factors (VIFs) were assessed to check multicollinearity among independent variables. A VIF below 10 was considered an absence of multicollinearity [33]. Two variables were correlated; “ANC utilisation” and “frequency of ANC utilisation”. Of these two collinear covariates, only “ANC utilisation” was retained in the multivariable analysis. Logistic regression was conducted to calculate crude and adjusted odds ratios (ORs) and 95% CIs. Variables with a *p*-value of less than 0.2 in the bivariate analyses were included in multivariable models. To avoid residual confounding in multivariable analysis, a p-value of less than 0.20 was considered adequate [34]. Three steps were used to assess how factors from various levels affected NNM. Independent variables were entered into the model progressively, from distant to the proximal factors. A three-step procedure was conducted to construct a multivariable model. In Model I, distant factors were included. In Model II, intermediate factors were added to Model I. In Model III, proximate factors were added to Model II. The best model was taken by obtaining log-likelihood ratio tests, Akaike’s information criteria (AIC) and adjusted-R^2^.

### Operational definitions

- **Neonatal near miss** as a neonate who nearly died but survived a severe complication that occurred before being discharged from hospital or seven postpartum days if the newborn stayed in the hospital.
- **Neonatal mortality** as a neonate who died before being discharged from the hospital or died within seven postpartum days if the newborn stayed in the hospital.

## Results

**Fig. 1:**
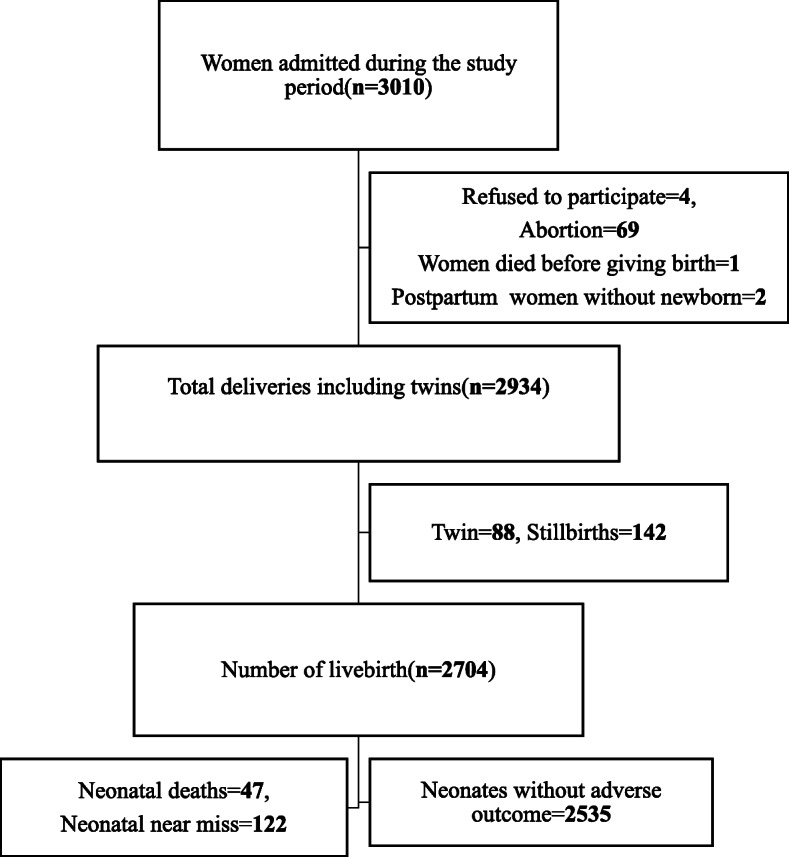
Study flow chart of neonatal near miss in south Ethiopia, 2018

There were 3010 women admitted to the selected hospitals during the study period. After excluding those who did not wish to participate [4], abortion cases (69), postpartum women [2], maternal death during pregnancy [1], twin pregnancies (88), and stillbirths (142) a total of 2704 live births were included in this study. Of the total 2704 live births, 2535 (93.8%) newborns did not exhibit any neonatal adverse outcomes (Fig. 1).

### Sociodemographic characteristics

More than half (51.6%) of the women in the study resided in an urban area. The mean age of the women was 26 (+ 4.6) years; the majority (83.3%) were aged 21–35 years. The majority (99.2%) of the women were married and most (61.2%) were Protestant. Half of both parents had received secondary education. More than two-thirds (70.6%) of the women were housewives and 37.7% of fathers were daily labourers. More than half (53.1%) of the women reported a monthly family income of less than < 121 USD (Table 2).

**Table 2:** Socio-demographic characteristics of the women in south Ethiopia, 2018

Variables	Number (%)
**Place of residence**	
Rural	1309 (48.4)
Urban	1395 (51.6)
**Age**	
< =20	384 (14.2)
21–35	2252 (83.3)
> 35	68 (2.5)
**Marital status**	
Married	2682 (99.2)
Single	16 (0.6)
Divorced / Widowed	6 (0.2)
**Religion**	
Protestant	1654 (61.2)
Ethiopian Orthodox	566 (20.9)
Muslim	345 (12.8)
Catholic	105 (3.9)
Others*	34 (1.3)
**Maternal education**	
Cannot read and write	117 (4.3)
Able to read and write but no schooling	140 (5.2)
Primary school	959 (35.5)
Secondary school	1364 (50.4)
College and above	124 (4.6)
**Maternal Occupation**	
Housewife	1909 (70.6)
Student	164 (6.1)
Merchant	181 (6.7)
Government employee	124 (4.6)
Daily labourer	307 (11.4)
Other**	19 (0.7)
**Paternal education**	
Cannot read and write	33 (1.2)
Able to read and write but no schooling	154 (5.7)
Primary school	882 (32.6)
Secondary school	1396 (51.6)
College and above	239 (8.8)
**Paternal occupation**	
Student	67 (2.5)
Merchant	649 (24)
Government Employee	241 (8.9)
Farmer	483 (17.9)
Daily labourer	1018 (37.7)
Other***	244 (9.0)
**Monthly income**	
**< =**79 USD	612 (22.6)
80–121 USD	824 (30.5)
122–155 USD	621 (23)
> 155USD	647 (23.9)

* Adventist, Hawariyat ** farmer, private business *** Driver, Tailor, Church leader, Guard, Private business

### Reproductive characteristics

Nearly half (46.9%) the women had at least one living child and the majority (88.1%) gave birth at term (37–42 weeks). More than half (52.4%) the women had their first pregnancy at the age of ≤20 years; 9% had experienced abortion and 5.9% had had stillbirths. Forty-five percent of the women became pregnant in or after two years of their preceding pregnancy. The majority (94.2%) of the women reported their pregnancy as a planned pregnancy. The majority (93.7%) had at least one ANC visit. However, most (81.5%) started after the recommended time (after 12 weeks of gestation). More than half (56.1%) of the women attended only one to three visits. More than three-quarters (76%) of the women gave vaginal birth and more than one-quarter (27.9%) were referred from other health facilities. (Table 3).

**Table 3:** Reproductive characteristics of the women in south Ethiopia, 2018

Variables	Number (%)
**Number of children**	
1	1260 (46.9)
2–4	1258 (46.9)
> =5	167 (6.2)
**Gestational age**	
29–36	266 (9.8)
37–42	2383 (88.1)
> 42	55 (2)
**Age at first pregnancy**	
< =20	1417 (52.4)
21–25	1059 (39.2)
26–30	208 (7.7)
> 30	20 (0.7)
**Abortion**	
Yes	255 (9.4)
No	2449 (90.6)
**Stillbirth**	
Yes	160 (5.9)
No	2544 (94.1)
**Birth interval**	
< 24 months	357 (13.2)
> =24 months	1204 (44.5)
Not applicable	1143 (42.3)
**Type of pregnancy**	
Planned	2547 (94.2)
Unplanned	157 (5.8)
**Antenatal care utilization**	
Yes	2534 (93.7)
No	170 (6.3)
**Gestational age at first visit**	
< =12 weeks	468 (18.5)
> 12 weeks	2066 (81.5)
**Number of ANC visits**	
1–3	1421 (56.1)
> =4	1113 (43.9)
**Mode of delivery**	
Vaginal delivery	2056 (76.0)
Caesarean section	612 (22.6)
Instrumental delivery	36 (1.3)
**Referral from other health facilities**	
Yes	754 (27.9)
No	1950 (72.1)

### Neonatal near miss criteria

Seventy-five newborns presented with only the pragmatic criteria, while 43 displayed both pragmatic, clinical and management criteria. The remaining 4 presented with only clinical and management criteria. There were 118 neonates who met the pragmatic criteria for NNM, corresponding to an incidence of 43.6 per 1000 live births. An Apgar score of less than seven was the most common criterion identified for NNM, with an incidence ratio of 39.2 per 1000 live births. During the study period, 47 newborns died, resulting in an NMR of 17.4 (95% CI = 13.0–23.3) per 1000 live births. All 47 newborns who died met the pragmatic criteria, giving an incidence ratio of 17.4 per 1000 live births. As for NNM, an Apgar score was the most common identified criterion (46.9%), resulting in an NMR of 17 deaths per 1000 live births (Table 4).

**Table 4:** Criteria for identifying neonatal near miss in south Ethiopia, 2018

Criteria	NNM (%)	Incidence of NNM/1000 live births	NM (%)	Incidence of NMR per 1000 live births
**Pragmatic criteria**	118 (53.3)	43.6	47	17.4
Gestational age (weeks) < =33	59 (35.3)	21.8	35 (35.7)	12.9
APGAR Score < 7	106 (63.5)	39.2	46 (46.9)	17.0
Birthweight (g) < =1750	2 (1.2)	0.7	17 (17.3)	6.3
**Clinical and Management criteria**	45	16.6	39	14.4
Respiratory rate > 70/min	1 (2.2)	0.4	6 (6.3)	2.2
Cyanosis	19 (42.2)	7.0	22 (23.2)	8.1
Absence of regular breathing	0 (0)	0	24 (25.3)	8.9
Any intubation	3 (6.7)	1.1	2 (2.1)	0.7
Cardiac arrest	2 (4.4)	0.7	2 (2.1)	0.7
Cardiopulmonary resuscitation	9 (20)	3.3	12 (12.6)	4.4
Persistent bradycardia < 80 bpm	1 (2.2)	0.4	0 (0)	0
Persistent tachycardia > 200 bpm	4 (8.9)	1.5	1 (1.1)	0.4
Use of vasoactive drug	0 (0)	0	1 (1.1)	0.4
Blood transfusion	6 (13.3)	2.2	2 (2.1)	0.7
Use of anticonvulsant	–	–	–	–
Inability to suck within 12 h	15 (33.3)	5.5	5 (5.3)	1.8
Visible jaundice in first 24 h	5 (11.1)	1.8	7 (7.4)	2.6
Phototherapy	6 (13.3)	2.2	6 (6.3)	2.2
Any active non-traumatic bleeding	1 (2.2)	0.4	0	0
Haematuria	–	–	–	–
Anuria > 24 h	–	–	–	–
Apathetic, poor tolerance of feeds within 12 h	2 (4.4)	0.7	2 (2.1)	0.7
Abdominal distension and vomiting	–	–	–	–
Parenteral antibiotics	9 (20)	3.3	3 (3.2)	1.1

### Neonatal near miss indicators

The NNMR was 45.1 (95% CI = 37.7–53.8) per 1000 live births. The SNOR was 62.5 (95% CI = 53.8–72.5) per 1000 live births and the MI was 27.8%. Of those newborns who developed NNM, more than half (59.8%) of their mothers were referred from other health facilities. Of those newborns who died, 63.8% of their mothers were referred from other health facilities (Table 5).

**Table 5:** Indicators of neonatal near miss in south Ethiopia, 2018

Indicators	Value
Neonatal deaths	47
Neonatal Near miss	122
Severe neonatal outcomes	169
Neonatal mortality rate	17.4
Neonatal near miss rate	45.1
Severe neonatal outcome rate	62.5
Neonatal near-miss mortality ratio	2.6:1
Mortality index	27.8%
Near Miss Neonates of mothers referred from other health facilities	73
Percentage of Near Miss Neonates of mothers referred from other health facilities	59.8%
Newborn died of mothers referred from other health facilities	30
Percentage of Newborn died of mothers referred from other health facilities	63.8%

### Neonatal near miss and neonatal mortality among selected hospitals

The incidence of NNMR ranged from 25.4 per 1000 live births at DGH to 67.2 per 1000 live births at HUCSH. HUCSH has the highest NNMR and NMR. While NEMGH has the highest near miss to mortality ratio, which is 5.3:1.0 (Table 6).

**Table 6:** Incidence of neonatal near miss and neonatal mortality in three selected hospitals in south Ethiopia, 2018

	Durame General Hospital	Hawassa University Comprehensive Specialized Hospital	Nigist Eleni Mohammed General hospital	Total
Live births	669	1012	1023	2704
Neonatal near miss	17	68	37	122
Neonatal near miss incidence rate per 1000 livebirths (95% CI)	25.4 (15.3–41.2)	67.2 (52.9–84.9)	36.2 (26.0–50.1)	45.1 (37.7–53.8)
Neonatal deaths	7	33	7	47
Neonatal mortality incidence rate per 1000 livebirths (95%CI)	10.5 (4.6–22.5)	32.6 (22.9–46.0)	6.8 (3.0–14.7)	17.4 (13.0–23.3)
Severe neonatal outcomes	24	101	44	169
Severe neonatal outcomes incidence rate per 1000 live births(95% CI)	35.9 (23.6–53.7)	99.8 (82.4–120.3)	43.0 (31.8–57.8)	62.5 (53.8–72.5)
Near miss to mortality ratio	2.4:1.0	2.1:1.0	5.3:1.0	2.6:1.0

### Determinants of neonatal near miss

Table 7 shows the results of bivariate logistic regression analyses with the model fit statistics**.** In Model I (adjusted for individual factors), place of residence, maternal and paternal educational status, paternal occupation and monthly income showed a statistical association. In Model II, maternal and paternal educational status, monthly income, birth interval, type of pregnancy, ANC utilisation and referral status showed a statistically significant association with NNM. After addition, the proximal factors in Model III (controlling for all variables), monthly income, birth interval, and severe maternal complications remained significantly associated with NNM. Monthly income was the distant factor associated with NNM. Newborns whose mothers had a monthly income of less than 79 USD were more likely to develop NNM than those with a monthly income greater than 79 USD (AOR: 2.34; CI: 1.10–4.99). Newborns born within a preceding interval of less than 24 months had a higher likelihood of experiencing NNM than those born at an interval of 24 months or more (AOR:4.68; CI: 2.52–8.67). Newborns whose mothers had potentially life-threatening complications had a higher risk of experiencing NNM compared to those who did not have complications (AOR: 12.86; CI: 7.82–21.15).

**Table 7:** Determinants of neonatal near miss in south Ethiopia, 2018

Variables	Neonatal Near miss		UOR(95%CI)	Model-I	Model-II	Model-III
	Yes	No		AOR(95%CI)	AOR(95%CI)	AOR(95%CI)
**Distant factors**						
**Place of residence**						
Rural	73 (5.7)	1204 (94.3)	1.65 (1.14–2.39)	1.34 (0.91–1.99)	1.08 (0.70–1.65)	
Urban	49 (3.6)	1331 (96.4)	1	1	1	
**Maternal education**						
Primary and below	98 (8.3)	1077 (91.7)	5.53 (3.51–8.70)	3.14 (1.93–5.10)	2.11 (1.27–3.52)	1.63 (0.94–2.84)
Secondary and above	24 (1.6)	1458 (98.4)	1	1	1	1
**Paternal education**						
Primary and below	83 (8)	951 (92)	3.55 (2.40–5.23)	1.72 (1.12–2.63)	1.44 (0.91–2.23)	1.32 (0.80–2.19)
Secondary and above	39 (2.4)	1584 (97.6)	1	1	1	1
**Maternal occupation**						
Housewife	100 (5.4)	1767 (94.6)	1.98 (1.24–3.16)	0.95 (0.57–1.57)		
Other	22 (2.8)	768 (97.2)	1	1		
**Paternal occupation**						
Farmer	45 (9.6)	425 (90.4)	2.90 (1.98–4.25)	1.58 (1.04–2.40)	1.50 (0.94–2.40)	1.08 (0.63–1.84)
Others	77 (3.5)	2108 (96.5)	1	1	1	1
**Monthly income**						
< =79	72 (12.5)	504 (87.5)	8.23 (4.32–15.70)	4.72 (2.41–9.25)	2.96 (1.45–6.03)	2.34 (1.10–4.99)
80–121	34 (4.2)	783 (95.8)	2.50 (1.26–4.98)	1.98 (0.98–4.01)	1.78 (0.89–3.71)	1.34 (0.61–2.92)
122–155	5 (0.8)	614 (99.2)	0.47 (0.16–1.36)	0.44 (0.15–1.27)	0.48 (0.16–1.41)	0.47 (0.15–1.43)
> 155	11 (1.7)	634 (98.3)	1	1	1	1
**Intermediate factors**						
**Birth interval**						
< 24 months	72 (22.2)	253 (77.8)	16.80 (10.05–28.06)		8.52 (4.88–14.88)	4.68 (2.52–8.67)
> =24 months	20 (1.7)	1180 (98.3)	1		1	1
First birth	30 (2.7)	1102 (97.3)	1.61 (0.91–2.85)		1.69 (0.94–3.70)	1.42 (0.77–2.66)
**Type of pregnancy**						
Planned	96 (3.8)	2411 (96.2)	1		1	1
Unplanned	26 (17.3)	124 (82.7)	5.27 (3.29–8.42)		1.97 (1.09–3.56)	1.54 (0.78–3.01)
**ANC service utilization**						
Yes	90 (3.6)	2414 (96.4)	1		1	1
No	32 (20.9)	121 (79.1)	7.09 (4.56–11.05)		2.05 (1.17–3.59)	1.65 (0.86–3.16)
**Frequency of ANC**						
1–3 visits	67 (4.8)	1326 (95.2)	2.39 (1.48–3.86)			
> =4 visits	23 (2.1)	1088 (97.9)	1			
**Women’s referred from other health facility**						
Referred	73 (10.1)	651 (89.9)	4.31 (2.97–6.26)		2.12 (1.38–3.27)	1.32 (0.81–2.16)
Not Referred	49 (2.5)	1884 (97.5)	1		1	1
**Proximal factors**						
**Mode of delivery**						
Vaginal delivery	70 (3.4)	1995 (96.6)	0.36 (0.25–0.53)			0.67 (0.40–1.10)
Caesarean section	52 (8.8)	540 (91.2)	1			1
**Infant sex**						
Female	44 (3.5)	1203 (96.5)	1			1
Male	78 (5.5)	1332 (94.5)	1.60 (1.10–2.34)			1.48 (0.93–2.37)
**Maternal complications**						
Yes*	78 (42.4)	106 (57.6)	40.62 (26.76–61.68)			12.86 (7.82–21.15)
No	44 (1.8)	2429 (98.2)	1			1
**Log-likelihood**			–	− 830	−700	− 587
**AIC**			–	1688	1440	1222
**Adjusted-R**^**2**^			–	0.188	0.333	0.453
**VIF**			–	1.23	1.50	1.83

**UOR-** Unadjusted odds ratio **AOR**- Adjusted odds ratio, **AIC**- Akaike Information Criterion, **VIF**- Variance Inflation Factor

The log-likelihood of NNM increased from Model I to Model III. Conversely, the AIC decreased from 1688 in Model I to 1222 in Model III. The average VIF of Model III was 1.83. These model fit statistics, including the adjusted-R^2^, indicate that Model III fits the data better than other models.

## Discussion

Evidence reported that NNM is important to assess and improve the quality of neonatal care [6, 10, 12]. However, there is no internationally agreed criteria for defining or identifying NNM. For instance, Pillegi-Castro et al. used both pragmatic and management criteria, while a study conducted in Brazil used only the pragmatic criteria to identify the NNM cases [9, 10]. Use of different criteria may result in underreporting or over-reporting of actual cases. Therefore, developing a uniform definition to identify NNM could minimize bias related to the selection of criteria.

In this hospital-based prospective cohort study, the burdens of NNMR and NMR were 45.1 (95% CI = 37.7–53.8) and 17.4 (95% CI = 13.0–23.3) per 1000 live births, respectively. The following factors were associated with a higher likelihood of NNM: lower monthly income, birth interval of less than 24 months and severe maternal complications (potentially life-threatening complications). To the best of the authors’ knowledge, this study is the first to describe the determinants of NNM in Ethiopia.

The rate of NNM found in this study was lower than that in studies conducted in Uganda and Brazil, which showed NNMRs of 417 and 220 per 1000 live births, respectively [19, 26]. A possible explanation for the difference in these findings could be that studies conducted in Uganda and Brazil included only women with severe obstetric complications, unlike the present study. Another reason might be that the follow-up period in the present study was until hospital discharge or seven postpartum days if the newborn stayed in the hospital, while the study in Brazil followed the neonates until 28 days after birth.

The NNMR in the current study was 2.6 times higher than the NMR. Studies conducted in Uganda and Brazil reported that the NNMR varied from 2.1 to eight times higher than the NMR [9, 10, 19, 26]. Similarly, a worldwide systematic review on NNM indicated that the NNMR was 2.6 to eight times higher than the NMR [6]. Taken together, these studies indicate that for every neonate who dies, many others experience a severe complication that may lead to death or disability.

The NMR in the current study was 17.4 deaths per 1000 live births, which is lower than those found in a national report in Ethiopia and other studies conducted in the country [14, 17, 22]. The reason for this difference could be that in the previous studies, most study participants were from rural areas where women may have poor access to health care. Further, the inconsistencies may also be related to the time that has passed since the studies were undertaken, as the government has implemented strategies such as introducing newborn care practices, increasing midwifery professionals and expanding neonatal intensive care units in recent years. This suggestion is supported by other studies that have stated that Ethiopia has recently made remarkable progress regarding the reduction of under-five mortality (by 67%), reflecting the government’s commitment to scaling up interventions through disease control program (health extension program) and strengthening the health system [35, 36].

Studies revealed that NNM could be used as a measure of quality of care in a facility [8, 10, 12, 20] whereby a higher near miss to mortality ratio indicates a better quality of care. The current study indicated that the incidence of the NNM varied among hospitals, which is 25.4, 67.2 and 36.2 in DGH, HUCSH, and NEMGH, respectively. The teaching hospital (HUCSH) showed a higher incidence of NNMR compared to the two regional hospitals (DGH and NEMGH). Further, the teaching hospital has a lower near miss to mortality ratio. The reason for this difference may be that the teaching hospital may receive more complicated cases than the regional hospitals. Therefore, it is not possible to conclude that the quality of health care is poorer in teaching hospitals compared to the regional hospitals.

According to the World Health Organization (WHO), if a large proportion of women arrive at health facilities with complications, this suggests the failure of the referral chain in the facilities [8]. The current study revealed that of those newborns who developed NNM, more than half (59.8%) of their mothers were referred from other health facilities. A study conducted in Ethiopia reported that delays created at home or in health facilities were the major factor contributing to neonatal deaths [37]. This finding is supported by studies conducted in Africa that stated that delays in seeking and reaching maternal care increased the risk of neonatal complications and deaths [38, 39]. This highlights the need to strengthen the referral status of facilities to reduce neonatal mortality.

Neonates born to mothers who had a low monthly income were identified as at risk for NNM in the current analysis. This finding is supported by previous studies conducted in low- and middle-income countries [32, 40, 41] and south-west Ethiopia [21]. The reason for this might be that women with high incomes may have better access to health care services and better living conditions. A study of neonatal survival reported that poor household income increased neonatal morbidity and mortality either by reducing access to effective and quality care or by increasing the rate of infection [42]. Demographic and Health Survey data from sub-Saharan African and South Asian countries indicated that reducing the mortality gap between the richest and poorest mothers could avert 750,000 newborn deaths [42]. This implies that addressing inequity may be one strategy for improving survival and reducing the morbidity of newborns.

The current study found that neonates born with a birth interval of less than 24 months were more likely to develop NNM than those born at an interval of 24 months or more. Similar to these findings, previous studies conducted in low- and middle-income countries reported an association of neonatal death with birth intervals less than 24 months [22, 43, 44]. A study undertaken in Bangladesh reported that as the birth interval increases up to a minimum of 24 months, the risk of early neonatal deaths decreases [45]. Further, a three-year analysis of the EDHS report indicated that neonates born within a preceding birth interval of less than 24 months were two times more likely to die than those born at an interval of 24 months or more [22]. The short birth interval effect in neonates may be related to maternal nutritional depletion, mostly due to the physiological competition between the mother and the growing foetus. Prolongation of birth interval between pregnancies helps the mother to prepare for the future pregnancy [46, 47]. Together, these findings suggest that promoting postpartum family planning might reduce the rate of neonatal complications and deaths.

This study’s results found that ANC utilisation had no significant association with NNM, which is inconsistent with the findings of previous studies from Ethiopia and other countries [48–51]. Studies conducted in Brazil revealed that ANC utilisation during pregnancy reduces the risk of NNM [18, 19]. The reason for the difference in these findings may be that those women who utilised ANC in the present study may not have received quality care. This is supported by a Ministry of Health report (2015) that stated that the proportion of pregnant women who received ANC at least once surpassed 98% [36]. However, the continuity of the services and quality of care was suboptimal, as evidenced by low tetanus toxoid vaccine uptake, micronutrient supplementation and uptake of prevention of mother-to-child transmission of HIV services by pregnant women [36]. Further, most women did not attend the recommended number of ANC visits [14]. This suggests that although increasing utilisation is important, a shift in focus from increasing utilisation of ANC to improving the quality of care is required to achieve the global targets for newborn survival.

The current study found that severe maternal complication was a strong predictor of NNM. This finding is consistent with previous studies conducted in sub-Saharan Africa countries [23, 26, 40, 49]. A study regarding adverse perinatal outcome in Ethiopia reported that neonates born to mothers who had severe complications were 10 times more likely to have a low Apgar score, eight times more likely to have a low birth weight and five times more likely to be admitted to a neonatal intensive care unit than neonates born to mothers who had no complications during delivery [23]. In contrast, a study conducted on NNM in Brazil reported that severe maternal complications have no association with NNM [19]. An explanation for these differences might be that the Brazilian study collected data from women with high-risk pregnancies. The findings of the present study suggest the need for special care and appropriate management during pregnancy and delivery for women who have potentially life-threatening complications to reduce the NNM and death.

This study has notable strengths. A gap in the literature exists based on the shortage of studies that assessed NNM using the three criteria (pragmatic, clinical and management) [6, 26]. The current study has added to the literature by assessing the determinants of NNM by using pragmatic, clinical and management criteria. Another strength of the study is that all the mothers and their neonates were followed prospectively until hospital discharge or seven postpartum days if the newborn stayed in the hospital. Further, three steps were used to assess how factors from various levels affect NNM. However, the findings of the study must be interpreted in light of the following limitations. The study was facility-based and may not be representative of the wider population. Data were only collected up to discharge of mothers and newborns from the hospitals and, therefore, cases occurring after discharge may have been missed. Future research should be conducted to encompass the full 28-day postnatal period. The current study did not assess the quality of ANC as it was beyond the available resources. However, the findings suggest that an assessment of ANC quality might be warranted since in the current study, attendance of ANC visits did not show a statistical association.

## Conclusion

This study showed that low monthly income, birth interval less than 24 months and severe maternal complications increase the risk of NNM. Strategies to improve neonatal survival need a multifaceted approach that includes socio-economic and health-related factors. The findings of this study highlight important implications for policymakers with regard to NNM. In particular, addressing inequalities by increasing women’s income, promoting an optimal birth interval of 24 months or above through postpartum family planning, and preventing maternal complications may improve newborn survival. Moreover, referral systems must be improved to improve neonatal survival. Further research is also needed to measure the burden of NNM throughout the whole neonatal period.

## Data Availability

Data essential for the conclusion are included in this manuscript. Additional data can be obtained from the corresponding author on a reasonable request.

## Abbreviations

AOR: Adjusted odds ratio
AIC: Akaike Information Criterion
CI: Confidence interval
NNM: Neonatal near miss
NNMR: Neonatal Near Miss Rate
